# Quality Attributes and Dielectric Properties of Sea Buckthorn Berries under Differing Freezing Regimes and Their Interrelationships

**DOI:** 10.3390/foods11233825

**Published:** 2022-11-27

**Authors:** Moruo Li, Jingming Hu, Mei Yang, Jinfa Yang, Qianglin Zhang, Yury A. Zubarev, Wuyun Zhao, Yang Bi

**Affiliations:** 1 College of Mechanical and Electrical Engineering, Gansu Agricultural University, Lanzhou 730070, China; 2 Federal Altai Scientific Center of Agro-Biotechnologies, 35 Nauchniy Gorodok, Barnaul 656910, Russia; 3 College of Food Science and Engineering, Gansu Agricultural University, Lanzhou 730070, China

**Keywords:** sea buckthorn, frozen storage, quality attribute, dielectric property, prediction model

## Abstract

Fruit quality attributes interrelate with their dielectric properties, but such interrelationships in sea buckthorn berries under differing freezing regimes remain uninvestigated. Sea buckthorn (*Hipophae rhamnoides* L., cv. Shenqiuhong) berries were frozen at different temperatures (−13, −30, −35 and −40 °C) and stored for different periods (15, 30, 45, 60, 75 and 90 d). Seven quality attributes and nine dielectric parameters were measured to evaluate the effect of different frozen storage regimes on those attributes and parameters. The results showed that shorter time and lower temperature contributed to the preservation of berries quality. The dielectric parameters values increased with decreasing temperature and with the increase of freezing duration. The quality prediction models were established by the principal component analysis of the dielectric properties at characteristic frequency. The results are expected to provide a way to evaluate quality of frozen sea buckthorn berries by dielectric properties.

## 1. Introduction

Sea buckthorn (*Hippophae rhamnoides* L.) is a shrub of the *Elaeaceae* family. Its berries were often postharvest preserved under freezing before processing, but the berry quality reduced with the storage extension [1]. Cryopreservation was an effective way to preserve perishable fruit [2], due to the fact that the content of total phenolic and anthocyanins and the antioxidative activity of frozen haskap berries decreased significantly with the storage extension, and the lower the freezing temperature was, the more antioxidant components were preserved [3]. The content ascorbic acid of strawberries frozen at −27 °C for 90 d was significantly lower than that in the early freezing period [4]. Soluble solids and total phenolics of frozen blackberries decreased with the increasing of freezing duration [5]. The freezing duration also affected the titratable acids of grapes, and destroyed the structure of cells, with a 25% loss compared to fresh grapes [6]. Prolonging freezing storage duration not only compromised the fruit quality, but also affected their dielectric properties. Freezing at −18 °C destroyed the cell membrane of blueberries, resulting in the leakage of electrolytes and other compound extravasation, the decrease of complex impedance values, extracellular resistance and membrane capacitance, and low frequency impedance values were significantly higher than the high frequency complex impedance values. Due to the destruction of the cell membrane, the peel pigment of the fruit spread into the pulp, in which it deepened the pulp color. The complex impedance values could be used as an important indicator of cell membrane structure integrity by demonstrating a correlation between the membrane and the variations in the color and size of the frozen blueberries [7]. Freezing could destroy the cell membrane of sweet orange and increase the electrical conductivity of cells, resulting in significant decrease of complex impedance and capacitance values [8]. Additionally, fresh and frozen oranges could be differentiated using dielectric characteristics [9]. Freezing accelerated free water loss in strawberries, and the dielectric loss coefficient increased with increasing temperature [10]. Bian et al. [11] used the two principal components of ten dielectric parameters at the characteristic frequencies that established the physicochemical quality prediction models of bruised apples during storage. Dielectric properties could predict quality indices of kiwifruit during storage [12]. Dielectric properties could also be used as a method for determining SSC of persimmons [13]. There exists a mathematical relationship between the total soluble solids content and the impedance (Z), resistance (R), admittance (Y) and conductance (G) of apple juice, and these dielectric parameters could be used to determine the TSS of apple juice [14].

Although there have been studies on the changes of fruit quality attributes and dielectric parameters and their interactions during freezing, how the quality and dielectric properties of sea buckthorn berries change during freezing and their interrelationships have not been examined. In this study, the principal component and grey relation analysis were used to analyze the dielectric properties and quality attributes of sea buckthorn berries under different freezing time and temperature. The characteristic frequencies of the best dielectric parameter corresponding to each quality attributes were used to establish the detection model of the quality attributes.

## 2. Materials and Methods

### 2.1. Materials

Sea buckthorn berries (cv. Shenqiuhong) were harvested 120 d after flowering in 2021 from Ecological Industrial Park (38°43′26″ N, 100°39′36″ E), with an altitude of 1666 m in Minle County, Zhangye City, China. The berries were immediately packed and cooled with ice bags and transported to the College of Horticulture, Gansu Agricultural University.

### 2.2. Berry Processing and Freezing

Sea buckthorn berries of the same size and color, without mechanical damage and pest and disease infection, were selected. After the pedicel was cut manually, the berries were packed in polyethylene bags of (7 × 10 cm). In the control group, the sub-packaged berries were stored in a 4 °C ± 1 °C thermostatic humidifier (HWS-260B, Hangzhou Lubo Instrument Co., Ltd., Hangzhou, China). In the experimental group, the sub packaged berries were stored in a low-temperature refrigerator (BC/BD-200HER, Qingdao Haier Special Electric Co., Ltd., Qingdao, China) at −13 °C, −30 °C, −35 °C and −40 °C. The quality attributes and dielectric parameters of the berries were measured at 0 d, 15 d, 30 d, 45 d, 60 d, 75 d and 90 d, respectively, and the fresh fruits stored at 4 °C for 0 d were used as control.

### 2.3. Determination of Quality Attributes

#### 2.3.1. Chemicals

All chemicals used were of analytical grade. Potassium hydrogen phthalate, phenolphthalein, sodium hydroxide, anthrone, sucrose, ethyl acetate, gallic acid, aluminum chloride, concentrated sulfuric acid, sodium nitrite, anhydrous ethanol and sodium carbonate were purchased from Sinopharm Chemical Reagent Co. Ltd. (Shanghai, China). Catechin, ascorbic acid and oxalic acid were purchased from Shanghai McLean Biochemical Technology Co., Ltd. (Shanghai, China). Folinphenol was purchased from Beijing Soleibao Technology Co., Ltd. (Beijing, China). 2, 6-dichlorophenol indophenol was purchased from Shanghai Yuanye Biotechnology Co., Ltd. (Shanghai, China). The quality attributes were measured repeatedly three times.

#### 2.3.2. Determination of Water Content

The water content (WC) on wet bases of the berries was determined using a rapid moisture meter (QL-100A, Xiamen Qunlong Instrument Co., Ltd., Xiamen, China, accuracy ± 0.1%), according to the method of Huang et al. [15] and expressed as (%).

#### 2.3.3. Determination of Total Soluble Solids, Soluble Sugar, Titratable Acids and Ascorbic Acid

The content of total soluble solids (TSS), soluble sugar (SSC), titratable acids (TA) and ascorbic acid (AA) was determined by the procedure of Cao et al. [16].

Frozen sea buckthorn berries (5.0 g) were homogenized, centrifuged at 4000 r/min in a high-speed refrigerated centrifuge (3K15, SIGMA Laboratory Centrifuge, Osterode am Harz, Germany) for 10 min, at 4 °C, and the corresponding supernatants were collected. The content of total soluble solids (TSS) was determined by handheld refractometer (PAL-BXIACID2, ATAGO Co., Ltd., Tokyo, Japan) and expressed as (%).

The content of soluble sugar (SSC) was determined spectrophotometrically (UV-1780, Shimadzu Instruments (Suzhou) Ltd., Suzhou, China) using the anthrone-sulfate method with sucrose as standard. The absorbance of the mixture was measured at 620 nm and expressed as (%). The content of titratable acids (TA) was determined by sodium hydroxide solution titration method titrating with 0.1 mol/L NaOH to pH 8.1 and expressed as (%). The content of ascorbic acid (AA) was determined by the 2, 6-dichlorophenol-indophenol titration method titrating with calibrated 2, 6-dichlorophenol indophenol until it appears reddish and does not fade for 15 s and expressed as mg/100 g.

#### 2.3.4. Determination of Total Flavonoids and Total Phenolic Content

Frozen sea buckthorn berries (0.5g) were homogenized, extracted with 20 mL 75% (w/v) ethyl alcohol in a plug triangular bottle using a constant temperature shaker (TS-200B, Nanning Kechen Experimental Equipment Co., Ltd., Nanning, China) rotating oscillation at 120 r/min for 2 d at room temperature in darkness. Centrifuged at 5000 r/min using a high-speed refrigerated centrifuge (3K15, SIGMA Laboratory Centrifuge, Osterode am Harz, Germany) for 10 min at 4 °C, and the corresponding supernatants were collected.

Total flavonoids content (TFC) was determined spectrophotometrically (UV-1780, Shimadzu Instruments (Suzhou) Ltd., Suzhou, China) using the colorimetric method of Lay et al. [17] with some modifications. The extract (5 mL) was mixed with 2 mL of distilled water and 0.3 mL of a 5% NaNO2solution. After 5 min, 0.3 mL of a 10% AlCl3H2O solution was added, and after 1 min, 2 mL of 1 mol/L NaOH was also added to prepare the mixture. The solution was mixed well, and the absorbance was read at 510 nm.

Total phenolic content (TPC) was determined spectrophotometrically (UV-1780, Shimadzu Instruments (Suzhou) Ltd., Suzhou, China) using the Folin–Ciocalteu colorimetric method of Beato et al. [18] with some modifications. The extract (1.5 mL) was mixed with 2 mL of Folin–Ciocalteu reagent (10%; *w*/*v*) and 1 mL of NaCO3 (7.5%; *w*/*v*). The mixture was heated at 37 °C for 1 h in a water bath in darkness and the absorbance was measured at 760 nm.

Standard curves were established for flavonoids and phenolics contents using catechin (CE) and gallic acid equivalents (GAE) as standards. The results were expressed as CE mg/g FW and GAE mg/g FW.

### 2.4. Determination of Dielectric Parameters

**Figure 1:**
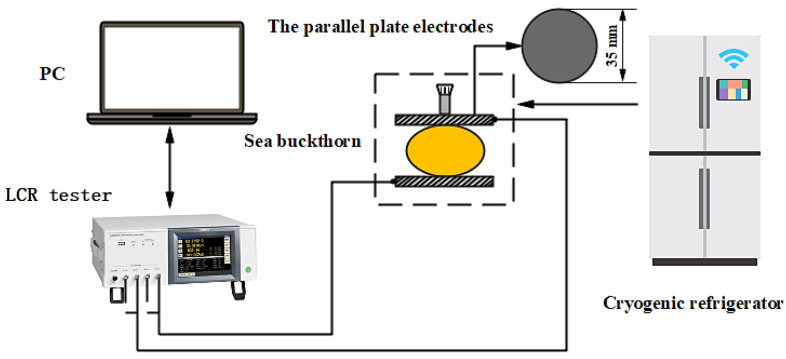
Experimental set up for measuring dielectric parameters of sea buckthorn.

Using the LCR tester [8] (IM3536, HIOKI (Shanghai) Measurement Technology Co., Shanghai, China), 300 low-frequency signal points of sea buckthorn berries was measured in the range of 1000 Hz and 1 MHz, the laboratory-made parallel electrode plate [19] was connected to the LCR tester, before the determination of dielectric parameters, we preheated the LCR tester for 1h. After the preheating, the open circuit (air) and short circuit correction parallel electrode plate were performed. After the correction, the dielectric parameters of the air were measured to determine whether it was necessary to correct again. The electrode plate fixture containing the sea buckthorn berries was placed in a refrigerator corresponding to the freezing temperature of the berries. The detection position was two symmetrical parts on the equator of the fruit, and the knob at the top of the parallel electrode plate was rotated to make the contact stable. The LCR tester was connected with a notebook computer by a USB data cable, and the dielectric parameters were measured and recorded online. The parallel electrode plate consists of two round copper plates (3 mm thickness × 35 mm diameter), and the electrode spacing was adjusted according to the size of berries. The test device is shown in Figure 1. The control temperature and humidity were constant during the test (temperature: −13 °C ± 0.6 °C, −30 °C ± 1 °C, −35 °C ± 0.6 °C, −40 °C ± 0.2 °C); RH: 65–70%). A total of nine dielectric parameters were measured as parallel equivalent capacitance (Cp), parallel equivalent resistance (Rp), complex impedance (Z), susceptance (B), conductance (G), quality factor (Q), parallel equivalent reactance (X), parallel equivalent inductance (Lp) and dielectric loss coefficient (D). The experiment was repeated five times.

### 2.5. Principal Component Analysis

Principal component analysis (PCA) is an analysis method that s (s ≤ p) unrelated common factors, as the comprehensive indicators, which can be obtained from the linear combination of p variables through the process of dimensional reduction. PCA transforms multiple potentially correlated variables which can be combined into a small number of uncorrelated composite variables called principal components [20]. The principal component analysis was performed on nine dielectric parameters of the berries at 300 detection frequencies, and stable principal components were chosen with a cumulative variance contribution greater than 80%. Before the principal component analysis, Z-score normalization was used to eliminate the effect of data dimensionality due to different units of the dielectric parameters.

### 2.6. Grey Relation Analysis

Grey relation analysis [21] is based on the degree of similarity between discrete data in the grey system to determine the size of the correlation and sort. The basic idea is to determine the degree of correlation between the various elements according to the similarity of the geometry of the feature sequence curve in the system. Grey relation analysis does not require many samples, and it can be applied to analyze irregular data [11]. The comparison sequence (subsequence) consisted of comprehensive parameters which represented the dielectric parameters of the frozen berries obtained by principal component analysis; the reference sequence (parent series) consisted of seven quality attributes of frozen sea buckthorn berries. The correlations between the quality attributes of frozen sea buckthorn berries and the principal components at each testing frequency were obtained by grey relation analysis, and the testing frequency with the highest correlation was selected as the characteristic frequency for predicting the quality attributes of the fruits.

### 2.7. Construction and Validation of Quality Attributes Prediction Models

Taking two principal components of the dielectric properties of the berries during freezing as independent variables, and the quality attributes as the dependent variables, the regression model was established to predict the quality attributes with the principal component of the dielectric properties. The prediction results were evaluated using the experimental data of sea buckthorn berries 90 d after the freezing as the validation group.

### 2.8. Statistical Analysis

The data were analyzed using IBM SPSS Statistics 25.0 software (Version 25.0, SPSS Inc., Chicago, IL, USA), and expressed as mean ± standard deviation (SD). The principal components of the dielectric parameters were selected by principal component analysis. The correlation between the characteristic frequencies of dielectric parameters and the quality attribute was determined by the Pearson correlation analysis (two-tailed test). Grey relation analysis of the dielectric parameter characteristic frequencies was performed using MATLAB R2019b software (The MathWorks Inc., Natick, MA, USA). Origin 2019b software (OriginLab Co., Northampton, MA, USA) was used to prepare graph and build predictive models for the quality attributes.

## 3. Results and Discussion

### 3.1. Effects of Freezing Time and Temperature on Quality of Sea Buckthorn Berries

**Figure 2:**
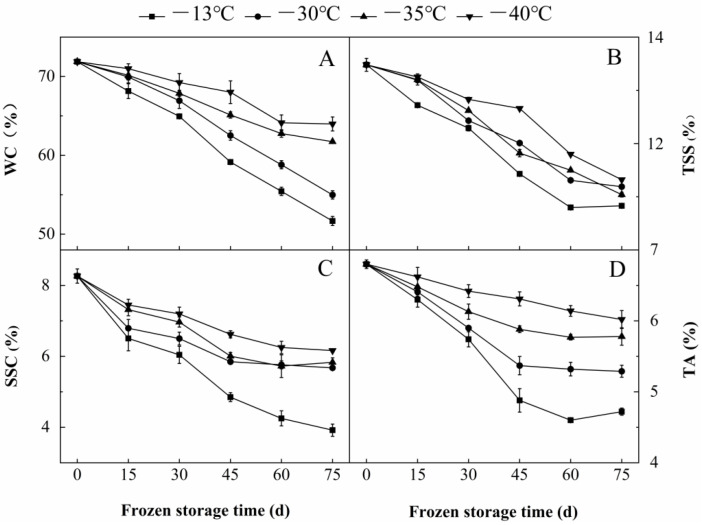
Effect of freezing time and temperature on the (**A**) WC, (**B**) TSS, (**C**) SSC and (**D**) TA of sea buckthorn berries. Bars indicated standard error (±SD). The -■-, -●-, -▲-, -▼- show the freezing temperature of −13, −30, −35 and −40°C, respectively. The data are mean values of three replicates.

WC, TSS, SSC and TA were important attributes which affected the quality of sea buckthorn berries [22]. With the extension of freezing time, the WC, TSS, SSC and TA contents of the berries steadily decreased, and frozen at −30 °C for 45 d were lower than those frozen at 0 d by 13.0%, 10.9%, 29.2% and 21.0%, respectively. The WC, TSS, SSC and TA contents of the berries at −30 °C for 75 d were lower than those frozen at 0 d by 23.5%, 17.0%, 31.3% and 22.2%, respectively (Figure 2). With the decrease in freezing temperature, the quality attributes of the berries also decreased steadily. The quality attributes of the fruit stored at −30 °C for 45 d were higher than those at −13 °C by 5.4%, 4.8%, 17.1% and 9.1%, respectively. The quality attributes of the berries frozen at −40 °C for 45 d were higher than those at −13 °C by 13.0%, 10.8%, 26.7% and 22.7%, respectively (Figure 2). With the extension of freezing time, the berry cells experienced compressive stress during the transformation of water into ice crystals, and the ice crystals endured tensile stress caused by cell shrinkage during further cooling. When the tensile stress exceeded the rupture stress of cell structure, the difference between the compressive stress and tensile stress resulted in cell rupturing [23]. This made the intracellular TSS, SSC and TA easier to spill and degrade, resulting in reduced contents [24]. Water sublimated slowly during freezing, resulting in the decrease in the WC of the berries. Compared with ambient water vapor, the water vapor pressure inside the berries was saturated during freezing, and with the decrease in freezing temperature, the ice crystals at low temperature were less likely to sublime into the freezing environment due to the presence of different pressures [25]. As temperature dropped, the berry respiration rate grew less and metabolic activity slowed down [26]. The consumption of TSS and SSC slowed down [27], and the decrease of enzyme activity affected the TCA cycle reaction speed [28]. Therefore, the lower the freezing temperature, the better the preservation effect of WC, TSS, SSC and TA of berries.

**Figure 3:**
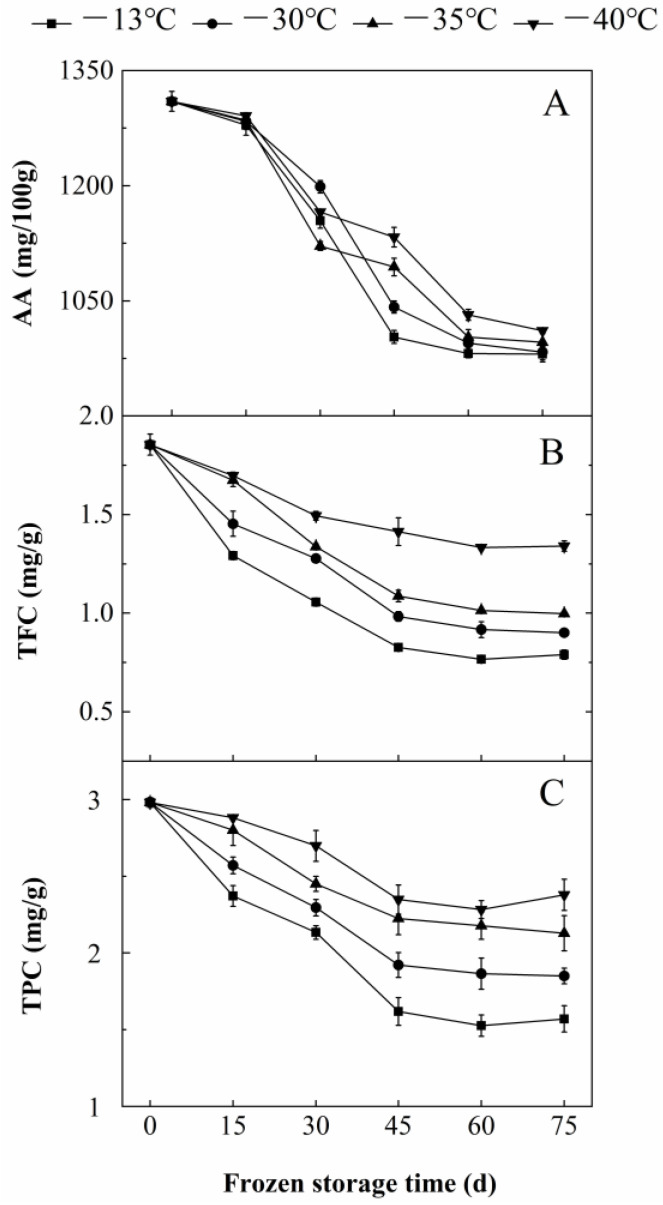
Effect of freezing time and temperature on the content of (**A**) AA, (**B**) TFC and (**C**) TPC of sea buckthorn berries. Bars indicated standard error (±SD). The -■-, -●-, -▲-, -▼- show the freezing temperature of −13, −30, −35 and −40 °C, respectively. The data are mean values of three replicates.

AA, TFC and TPC were important antioxidant components of sea buckthorn berries [29,30]. With the extension of freezing time, the contents of AA, TFC and TPC of the berries steadily decreased. The contents of AA, TFC and TPC of berries stored at −30 °C for 45 d were lower than those at 0 d by 20.5%, 47.0% and 35.5%, respectively. The contents of AA, TFC and TPC of the fruits frozen at −30 °C for 75 d were 24.9%, 51.4% and 38.0% lower than those at 0 d, respectively (Figure 3). As the freezing temperature decreased, the contents of AA, TFC and TPC of the berries decreased. The contents of AA, TFC and TPC of the berries at −30 °C for 45 d were 3.7%, 15.9% and 15.8% higher than those at −13 °C, respectively. The contents of AA, TFC and TPC of the berries at −40 °C for 45 d were lower by 11.5%, 41.5% and 31.1% than those stored at −13 °C (Figure 3). With prolonged freezing time, ice crystals could disassemble the structure of berry cell membrane, leading to the destruction of substrate and enzyme separation and the increase of contact probability [3]. AA was oxidized by ascorbic acid oxidase (AAO) [4]. TFC and TPC were easily oxidized by polyphenol oxidase (PPO) and peroxidase (POD) [31]. With the decrease in freezing temperature, the integrity of the fruit cell membrane was maintained [24], and the oxidation rates of AA, TFC and TPC decreased [32].

### 3.2. Effects of Freezing Time and Temperature on Cp, Rp and Z Values of Sea Buckthorn Berries

**Figure 4:**
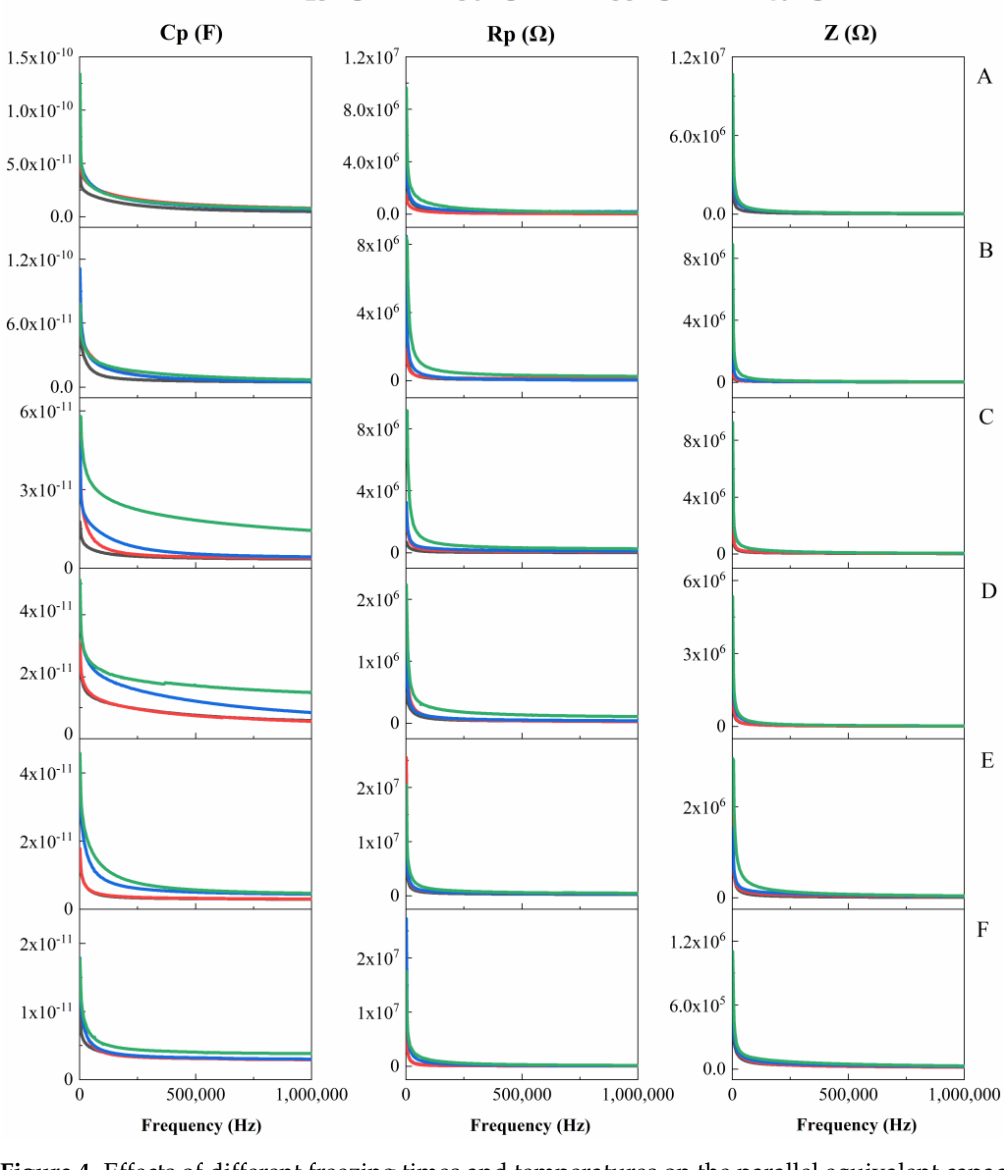
Effects of different freezing times and temperatures on the parallel equivalent capacitance Cp, parallel equivalent resistance Rp and complex impedance Z of sea buckthorn berries. ((**A**), 0 d; (**B**), 15 d; (**C**), 30 d; (**D**), 45 d; (**E**), 60 d; (**F**), 75 d). The data are mean values of five replicates.

The Cp and Z values of sea buckthorn berries decreased while the Rp value increased with the extension of freezing time. At 251,590 Hz, the Cp and Z values of the berries at −35 °C for 60 d were lower by 141.5% and 81.0% compared with the berries frozen at 0 d, respectively, while the Rp value was 49.6% higher than the berries frozen at 0 d. The Cp and Z values of berries frozen at −40 °C for 75 d were lower than those at 0 d by 244.1% and 6.2%, respectively, while the Rp value was 20.3% higher at 0 d (Figure 4). The Cp, Rp and Z values of sea buckthorn berries increased with the decrease in freezing temperature. Within the range of 1000–64,482 Hz, the Cp, Rp and Z values decreased rapidly, presenting an obvious linear relationship with the detection frequency. At 1000 Hz, the Cp, Rp and Z values of the berries at −40 °C for 30 d were 69.6%, 93.9% and 75.6% higher than those at −13 °C, respectively. In addition, the Cp, Rp and Z values of the berries at −35 °C for 75 d were 25.0%, 60.5% and 54.8% higher than those at −13 °C, respectively (Figure 4). The Cp, Rp and Z values of fruits decreased with the increase of detection frequency during freezing. The Cp, Rp and Z values at 1 MHz, frozen at −30 °C for 45 d were 4.47, 47.6 and 75.4 times lower than those at 1000 Hz, respectively (Figure 4). The Cp, Rp and Z values at low frequencies were significantly higher than those at high frequencies. This was because a low-frequency current passed through the extracellular fluid but not the cell structure. However, at high frequencies, some current passed through the cell membrane via intracellular fluid [33]. This phenomenon resulting from cell structures in biological tissue is known as β dispersion [34]. Therefore, it was speculated that the Cp, Rp and Z values of frozen berries decreased with the increase of detection frequency. The decrease of Z value with the increase of freezing time was caused by ice crystals puncturing the cell membrane, resulting in a decrease in the capacitance of the tissues [7].

### 3.3. Effects of Freezing Time and Temperature on B, G and Q Values of Sea Buckthorn Berries

**Figure 5:**
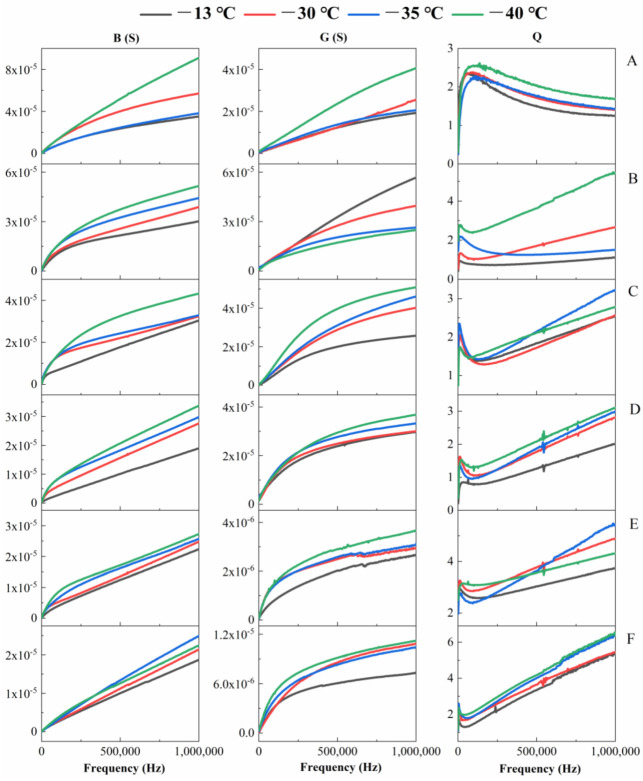
Effects of different freezing times and temperatures on the susceptance B, conductance G and quality factor Q of sea buckthorn berries. ((**A**), 0 d; (**B**), 15 d; (**C**), 30 d; (**D**), 45 d; (**E**), 60 d; (**F**), 75 d). The data are mean values of five replicates.

The B and G values of sea buckthorn berries decreased, while the Q value increased with the extension of freezing time. At 502,170 Hz, the B and G values of berries at −35 °C for 60 d were lower by 51.5% and 146.5% than those at 0 d, while the Q value was 51.4% higher than that frozen at 0 d. In addition, the B and G values of berries at −30 °C for 45 d were 155.9% and 10.7% lower than the berries frozen at 0 d, and the Q value was 3.8% higher than the berries frozen at 0 d (Figure 5). At the same detection frequency, the B, G and Q values of the berries increased with the decrease in freezing temperature. At 749,410 Hz, the B, G and Q values of berries stored at −40 °C for 75 d were 25.7%, 28.8% and 19.1% higher than those at −13 °C, respectively. The B, G and Q values of berries at −35 °C for 60 d were higher than those at −13 °C by 16.8%, 28.6% and 26.4%, respectively (Figure 5). The changes of B and G values of sea buckthorn berries during the freezing increased with the increase of detection frequency, and the Q value showed an increasing-decreasing-increasing trend with the increase of detection frequency (Figure 5). At low-frequency, the cell membrane capacitance is large, and the alternating current can only pass through the cell membrane. As the frequency increases, the capacitance of the cell membrane decreases, and the alternating current can pass through the entire protoplast [35,36]. With the decrease in freezing temperature and the extension of freezing time, owing to the effect of ice crystallization in extracellular fluids on tissue destruction [37], ice crystals destroyed the cell membrane structure, resulting in the leakage of intracellular water and soluble substances [38], the dielectric properties of berries changed. The permeability of the cell membrane was enhanced, and the bound water was converted to free water [39]. As the detection frequency increased, the ability and stability of acceptance current and conduction current of frozen sea buckthorn berries were enhanced.

### 3.4. Effects of Freezing Time and Temperature on X, Lp and D Values of Sea Buckthorn Berries

**Figure 6:**
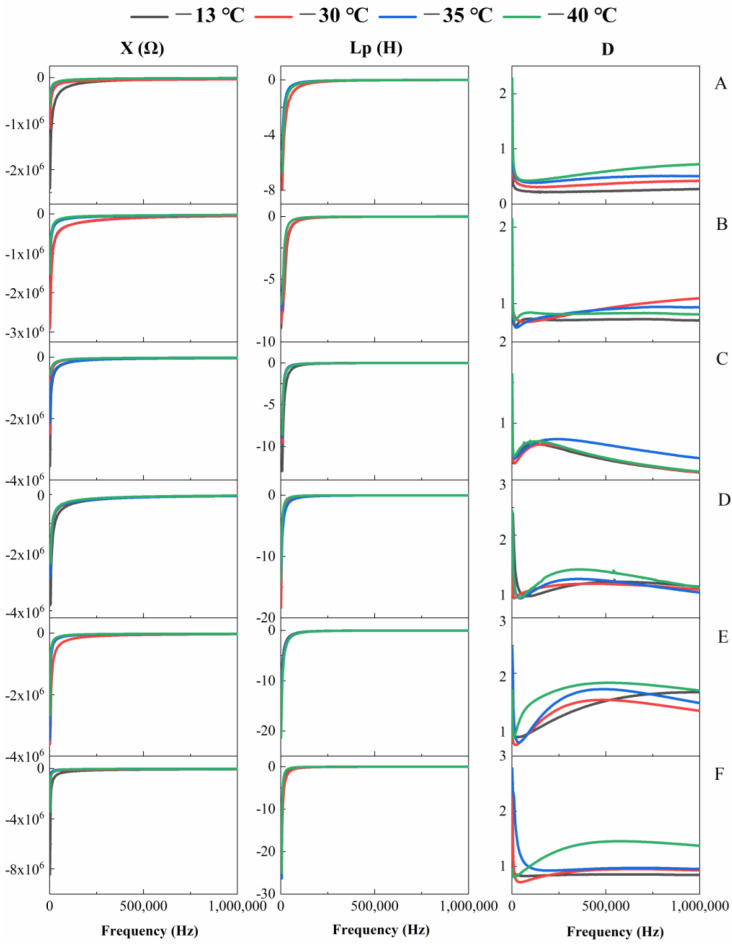
Effects of different freezing times and temperatures on the parallel equivalent reactance X, parallel equivalent inductance Lp and loss coefficient D of sea buckthorn berries. ((**A**), 0 d; (**B**), 15 d; (**C**), 30 d; (**D**), 45 d; (**E**), 60 d; (**F**), 75 d). The data are mean values of five replicates.

The X and Lp values of sea buckthorn berries decreased and the D value increased with the extension of freezing time. At 251,590 Hz, the X and Lp values at −30 °C for 60 d were 59.7% and 5.1% lower than those at 0 d, while the D value was 76.6% higher than 0 d. The X and Lp values of fruits stored at −35 °C for 45 d were 29.3% and 37.1% lower than those at 0 d. The D value was 64.5% higher than that at 0 d (Figure 6). At the same frequency, the X, Lp and D values of frozen berries increased with the decrease in freezing temperature. At 1000 Hz, the X, Lp and D values of berries held at −40 °C for 30 d were higher than those at −13 °C by 528.7%, 49.7% and 41.8%, respectively. The X, Lp and D values of berries at −40 °C for 45 d were higher than those at −13 °C by 22.9%, 18.2% and 14.6%, respectively (Figure 6). During the freezing, the X and Lp values of berries increased with the increase of detection frequency and then remained stable. The D value showed a trend of decrease-increase-decrease with the increase of detection frequency (Figure 6). The dielectric properties of the berries were affected by temperature [40]. With the decrease in freezing temperature, the ability of berries to produce electromagnetic induction increased [41]. The complex impedance value and the energy dissipation value in the electric field increased gradually. This was because the internal composition and the structure of the fruit were complex, which included various systems with different physical properties (cell membrane, cytoplasm, cytosol and bioelectrolyte). When these systems changed, their dielectric properties also changed [8]. Ando et al., [42] found that the damage of cell membrane led to a decrease of X value with the increase of temperature, the freezing caused cell membrane damage of sea buckthorn berries, resulting in the change of X value. In the low frequency area, the D value at 75 d of sea buckthorn berries was higher than 0 d, while in the high frequency area, the D values of sea buckthorn berries under differing freezing regimes were very similar, thus illustrating the dominant influence of ionic conduction at the lower frequencies and the dipolar losses at the higher frequencies [43,44]. The dielectric loss factor is related to various absorption mechanisms of energy dissipation [45], the D value showed a trend of decrease-increase-decrease with the increase of detection frequency, which might be caused by bound water and Maxwell–Wagner relaxations [13].

### 3.5. Principal Components of Dielectric Properties of Sea Buckthorn Berries during Freezing

**Figure 7:**
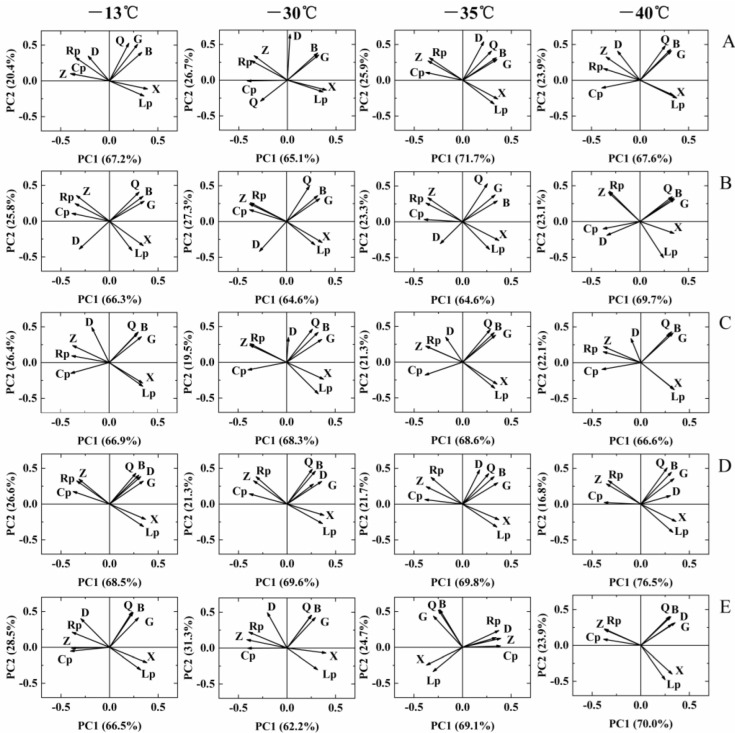
Principal component diagrams of dielectric parameters of sea buckthorn at different freezing temperatures and freezing times. ((**A**), 15 d; (**B**), 30 d; (**C**), 45 d; (**D**), 60 d; (**E**), 75 d).

After the principal component analysis of nine dielectric parameters of sea buckthorn berries during the freezing, two principal components (PCA1 and PCA2) were obtained according to the requirement that the principal component eigenvalue is greater than 1 (Figure 7). The variance contribution rates of PCA1 and PCA2 were 62.2–76.5% and 16.8–31.3%, the cumulative variance contribution of PCA1 and PCA2 was 87.6–95.0%. PCA1 and PCA2 could be used to characterize the dielectric properties of sea buckthorn berries during freezing. The information of Cp, Rp, Z, B, G and Q was mainly integrated in PCA1. The information of X, Lp and D was mainly integrated in PCA2.

### 3.6. Grey Relation Method to Select the Optimal Characteristic Frequency of Quality Attributes

The dielectric properties of sea buckthorn berries during freezing were closely related not only to their quality attributes, but also to the frequency of detection [12]. Therefore, in order to evaluate quality attributes with the dielectric properties, the detection frequencies that were more sensitive to the quality should be selected and then the best dielectric parameter corresponding to the quality could be determined. By calculating and comparing the correlation between the quality attributes and the dielectric parameters corresponding to each detection frequency, the detection frequency with the most significant correlation was selected as the characteristic frequency to predict the quality attributes of sea buckthorn berries. The result of correlation analyses between seven quality attributes and principal components of dielectric parameters is shown in Table 1. The results show that different quality attributes corresponded to different characteristic frequencies, and the characteristic frequencies were low frequencies [46]. This phenomenon is consistent with the theory that low-frequency current only passes through extracellular fluid.

### 3.7. Prediction Model of Quality Attributes of Sea Buckthorn Berries during Freezing

Different quality attributes correspond to different characteristic frequencies. At a characteristic frequency, the dielectric properties PCA1 and PCA2 can be used to predict the WC, TSS, SSC, TA, AA, TFC and TPC contents of frozen sea buckthorn berries. The quality prediction equation and determination coefficient are shown in Table 2. The determination coefficient of the TPC prediction equation was higher than 0.7, the determination coefficient of WC, SSC, TA, AA and TFC prediction equation was higher than 0.6, and the determination coefficient of the TSS prediction equation was higher than 0.5, indicating that the principal component of dielectric properties can be used to predict the seven attributes of frozen sea buckthorn berries.

### 3.8. Verification of Quality Prediction Equation

To verify the prediction accuracy of dielectric properties on WC, TSS, SSC, TA, AA, TFC and TPC contents, berries frozen for 90 d were used to compare the measured and predicted values of these seven quality attributes.

Table 3 shows the measured and predicted values of berries frozen for 90 d. The average relative error of TA, AA and TFC was higher than 10%, which indicated that the prediction accuracy was poor. The average relative errors of WC, TSS, SSC and TPC were less than 5%, indicating that the prediction accuracy was good.

## 4. Conclusions

The longer the freezing time and the lower the storing temperature, the faster the WC, the content of TSS, SSC, TA, AA, TFC and TPC of sea buckthorn berries decreased. At the same detection frequency, Cp, Z, B, G, X and Lp values of sea buckthorn berries decreased while the Rp, Q and D values increased with the increasing of freezing time. The values of dielectric parameters of the berries increased with decreasing temperature. As the detection frequency increased, the Cp, Rp and Z values decreased, the B and G values increased, the Q values showed an increasing-decreasing-increasing trend, the X and Lp values increased and then tends to be stable, and the D values showed a decreasing-increasing-decreasing trend. Because the dielectric properties of frozen sea buckthorn berries vary with different detection frequencies, we used grey relation analysis to select the characteristic frequency of principal components of dielectric properties with the maximum correlation degree. By using the principal components of the dielectric properties at the characteristic frequencies, a detection model for the WC, TSS, SSC, TA, AA, TFC and TPC content of frozen sea buckthorn berries was established. The average relative errors of WC, TSS, SSC and TPC contents were less than 5%. The prediction accuracy of the WC, TSS, SSC and TPC detection model was great, while the prediction accuracy of the TA, AA and TFC detection model was poor.

## Figures and Tables

**Table 1:** Correlations between seven quality attributes and dielectric parameters of sea buckthorn fruit during freezing.

Quality Attribute	Maximum Correlation of the First Principal Component (R2)	Characteristic Frequency of the First Principal Component (Hz)	Maximum Correlation of the Second Principal Component (R2)	Characteristic Frequency of the Second Principal Component (Hz)
WC	0.8010	635,820	0.5635	144,670
TSS	0.6958	615,700	0.6380	137,990
SSC	0.7336	602,400	0.5940	134,650
TA	0.8061	609,090	0.5959	127,960
AA	0.7452	612,430	0.6217	164,720
TFC	−0.7743	61,140	−0.5624	70,932
TPC	−0.8425	54,458	0.5595	127,960

**Table 2:** Correlations between seven quality attributes and dielectric parameters of sea buckthorn fruit during freezing.

Quality Attribute	Prediction Equation	Coefficient of Determination R^2^
WC	$y=32.0793+33{.27517x}_{1}+1{.99795x}_{2}$	0.644
TSS	$y=10.61726+3{.10878x}_{1}+0{.92816x}_{2}$	0.526
SSC	$y=5.99866+3{.78589x}_{1}+1{.9236x}_{2}$	0.648
TA	$y=2.99309+3{.5762x}_{1}+0{.37229x}_{2}$	0.659
AA	$y=252.68948+515{.54387x}_{1}\;-\;205{.42313x}_{2}$	0.643
TFC	$y=-0.14958\;-\;0{.46276x}_{1}\;-\;0{.07798x}_{2}$	0.622
TPC	$y=1.087\;-\;0{.58149x}_{1\;}+0{.41608x}_{2}$	0.746

Note: x_1_, x_2_ and y indicate PCA1, PCA2 and quality attribute.

**Table 3:** The measured and predicted values of fruit quality attributes of verified sea buckthorn group of frozen for 90 d.

Quality Attribute	−13 °C		−30 °C		−35 °C		−40 °C		Average Relative Error
	Measured	Predicted	Measured	Predicted	Measured	Predicted	Measured	Predicted	
WC	49.95 ± 0.11	53.76	54.43 ± 0.55	54.23	61.29 ± 0.13	59.88	63.58 ± 0.06	64.09	2.44%
TSS	10.50 ± 0.49	10.56	10.78 ± 0.06	11.12	11.00 ± 0.36	11.62	11.40 ± 0.19	11.88	3.39%
SSC	3.83 ± 0.48	3.56	4.89 ± 0.14	4.91	5.81 ± 0.55	5.88	6.40 ± 0.38	6.33	2.38%
TA	4.66 ± 0.06	2.99	4.87 ± 0.09	4.72	5.29 ± 0.11	4.78	5.86 ± 0.07	5.11	15.81%
AA	988.42 ± 3.68	1182.86	981.95 ± 2.74	979.65	996.06 ± 2.09	923.17	1010.53 ± 2.09	874.79	10.16%
TFC	0.60 ± 0.04	0.82	0.76 ± 0.03	0.74	0.90 ± 0.01	0.75	1.18 ± 0.25	0.71	24.01%
TPC	1.26 ± 0.05	1.26	1.60 ± 0.08	1.60	2.10 ± 0.01	1.96	2.25 ± 0.04	2.07	4.81%

## Data Availability

Data is contained within the article.
